# Mental Health Following Acquisition of Disability in Adulthood—The Impact of Wealth

**DOI:** 10.1371/journal.pone.0139708

**Published:** 2015-10-07

**Authors:** Anne Marie Kavanagh, Zoe Aitken, Lauren Krnjacki, Anthony Daniel LaMontagne, Rebecca Bentley, Allison Milner

**Affiliations:** 1 Gender and Women’s Health, Centre for Health Equity, Melbourne School of Population and Global Health, the University of Melbourne, 207 Bouverie St, Carlton, 3010 VIC, Australia; 2 Population Health Strategic Research Centre, School of Health & Social Development, Deakin University, Building BC3.213, 221 Burwood Highway, Burwood, VIC 3125, Australia; 3 McCaughey VicHealth Centre for Community Wellbeing, Centre for Health Equity, Melbourne School of Population and Global Health, the University of Melbourne, 207 Bouverie St, Carlton, 3010 VIC, Australia; University of Perugia, ITALY

## Abstract

**Background:**

Acquisition of a disability in adulthood has been associated with a reduction in mental health. We tested the hypothesis that low wealth prior to disability acquisition is associated with a greater deterioration in mental health than for people with high wealth.

**Methods:**

We assess whether level of wealth prior to disability acquisition modifies this association using 12 waves of data (2001–2012) from the Household, Income and Labour Dynamics in Australia survey–a population-based cohort study of working-age Australians. Eligible participants reported at least two consecutive waves of disability preceded by at least two consecutive waves without disability (1977 participants, 13,518 observations). Fixed-effects linear regression was conducted with a product term between wealth prior to disability (in tertiles) and disability acquisition with the mental health component score of the SF–36 as the outcome.

**Results:**

In models adjusted for time-varying confounders, there was evidence of negative effect measure modification by prior wealth of the association between disability acquisition and mental health (interaction term for lowest wealth tertile: -2.2 points, 95% CI -3.1 points, -1.2, p<0.001); low wealth was associated with a greater decline in mental health following disability acquisition (-3.3 points, 95% CI -4.0, -2.5) than high wealth (-1.1 points, 95% CI -1.7, -0.5).

**Conclusion:**

The findings suggest that low wealth prior to disability acquisition in adulthood results in a greater deterioration in mental health than among those with high wealth.

## Introduction

Nearly twenty percent of Australians report a disability, a prevalence similar to other developed countries [1,2]. People with disability are one of the most socio-economically disadvantaged groups in society [1]. In Australia, as in most other developed countries, people with disability have lower rates of employment, post-secondary education, income [3], and lower wealth [4]. Disabled people have poorer physical and mental health than those without disability, and many of the conditions they experience are unrelated to their impairment (e.g. diabetes and depression for people with intellectual impairments) [2]. There is some evidence to suggest that the relatively poorer health of people with disability may be partly explained by their disadvantaged living circumstances [5–7]. What is not known is whether the health consequences of acquiring a disability are influenced by access to pre-existing social, cultural or economic resources.

A number of studies have found an increase in psychological distress, depressive symptoms, and a reduction in subjective wellbeing following the acquisition of disability [8–10]. However, not all people who acquire a disability experience deterioration in their mental health and wellbeing [7]. The impacts of negative life events, such as the acquisition of a disability, on mental health may depend on individual resources such as personal resilience [11,12], experience of economic hardship [13], education [9], and social resources. While research on potential modifiers of the relationship between onset of a disability and mental health is scant, an Australian study of 136 young Australians showed that social and economic disadvantage prior to the onset of disability, including low levels of social support, was associated with poorer mental health following acquisition of disability [7]. A US longitudinal study of 478 adults found having above median wealth was associated with smaller decline in subjective wellbeing following acquisition of disability [14].

There is some evidence to show that inequalities in wealth within countries may contribute to health inequalities [11,15–18], particularly in the elderly [15,19]. While studies suggest that the association is independent of income and employment [17,20,21], the extent to which wealth is a causally associated with health is debated [22,23] as wealth may be a cause as well as a consequence of health [21]. Aside from having a direct effect on health, wealth may protect people who experience negative life events from deteriorations in health. Acquiring a disability in adulthood may lead to concerns regarding loss in future earnings because of being unable to work or needing to work fewer hours (potentially in less demanding, lower paid jobs). Wealth may provide a ‘safety net’ alleviating some of these concerns. Evidence from the Household, Income and Labour Dynamics in Australia survey (HILDA), on which this study is based, found that people who acquire a disability in adulthood were more likely to become unemployed or under-employed (i.e. employed in jobs for which they are over-educated and over-skilled) [24,25]. Wealth may also enable access to other resources, such as health and rehabilitative services, which could have mental health benefits.

The aim of this study is to assess whether pre-existing wealth moderates changes in mental health associated with acquisition of disability. The analysis includes nearly 2000 people who acquired a disability (at least two consecutive waves without disability followed by two consecutive waves with disability) in HILDA—a population-based panel survey of approximately 27 000 adults aged 15 years and older.

## Methods

### Data source

HILDA is a longitudinal nationally representative study of Australian households and individuals which includes data on range of life domains including social, demographic, health and economic characteristics. HILDA has been conducted in annual waves since 2001. The original panel included 13 969 individuals from 7682 households, sampled using a national probability sample of private dwellings [26]. Data were collected on each household member, and face-to-face interviews were sought from all household members aged 15 years or above. In later waves, survey members included all participants from the original panel and household members attaining the age of 15 from the original panel, with new participants added as a result of changes in household composition if new households were formed by existing survey participants. Response rates for the survey are above 90% for continuing participants and 70% for new participants invited into the survey.

### Outcome variable

The Mental Component Summary (MCS) score is derived from the Short Form 36 (SF–36) health survey. The SF–36 is a widely used self-completion measure of health status that has been validated for use in the Australian population, and to detect within-person changes in health over time [27]. The MCS represents a summary measure of mental health and wellbeing and is derived from factor analysis of the eight subscales in the SF–36, with the highest factor loadings for four subscales: mental health, role emotional, vitality and social functioning. The mean score on the MCS across all twelve waves (2001 to 2012) in the total HILDA sample was 48.8 (standard deviation 10.3).

### Disability measures

Information on long-term health conditions and disabilities was collected in all waves using a definition derived from the International Classification of Functioning, Disability and Health (ICF) framework [28]. Participants were asked if they had an ‘impairment, long-term health condition or disability which restricts their everyday activities that had lasted, or was likely to last, for a period of six months or more’.

Participants were defined as having acquired a disability if they did not report a disability for two consecutive waves followed by two consecutive waves with a reported disability. We used two waves so as to exclude people with transient disability and to reduce the potential for measurement error. If participants reported more than one episode of disability acquisition only the first episode was included. All consecutive waves in which individuals did not report a disability prior to disability acquisition and all consecutive waves reporting a disability subsequent to disability acquisition were included.

### Wealth

A wealth module was administered in 2002, 2006, and 2010. The module was constructed by the Reserve Bank of Australia and the Melbourne Institute of Applied Social and Economic Research. Most of the questions about assets and debts were asked at the household level and answered by one person on behalf of the household. Questions covered housing, incorporated and unincorporated businesses, equity-type investments (e.g., shares) and cash-type investments (e.g., bonds), and vehicles and collectibles (e.g., art works). Individuals were also asked questions about assets and debts, including superannuation, bank accounts, credit cards, student and other personal debts. While respondents were asked to give exact dollar amounts, bands were offered to those who could not provide an exact estimate of their superannuation holdings. More detail on this module has been published [29].

Household wealth (equivalent to the term net wealth) is a summary measure of total assets minus total debt for each household using the individual and household measures of assets and debt. Approximately 42% of the sample had at least one of the items used in the overall wealth variable missing. Missing information was most common for superannuation and business holdings [29]. Because of the inclusion of many types of assets and debt, most studies of wealth have a high proportion of missing data. Missing wealth data were imputed by the Reserve Bank of Australia using nearest neighbour imputation [29]. As this was a household measure, all household members were assigned the same value. Wealth data for 2006 and 2010 used longitudinal imputation at both the person- and household-level using the Little and Su imputation method [30].

The closest wealth record in waves preceding disability acquisition was used to measure prior wealth. The wealth variable was categorised into tertiles of the total HILDA distribution for each year.

### Other variables

Age was collapsed into four categories: under 30, 30–44, 45–59 and 60 years and above. Information on labour force status and occupational skill level was combined into a measure of employment that we have used previously [31] with five mutually-exclusive categories: unemployed (those who are actively seeking employment or currently unable to find work), not in the labour force (not actively seeking employment, for various reasons including education, retirement, infirmity/disablement, or household duties), low skill job (sales workers; machinery operators and drivers; labourers), medium skill job (technicians and trades workers; community and personal service workers; clerical and administrative workers) and high skill job (managers; professionals). Household disposable income was calculated by summing the income components for all adults in the household, with imputed values computed for missing variables using the methods described above (20% imputed values for observations in the sample) [29,30]. Household disposable income was equivalised using the modified OECD scale [32] and converted to national quintiles using statistics published annually by the Australian Bureau of Statistics [33].

### Statistical analysis

Twelve waves of HILDA data were included in the analysis (2001–2012). MCS scores are presented by wealth, age, sex, employment status and equivalised household income by disability status. Comparisons are made within-individuals with each individual having at least two waves without followed by two waves with disability. Therefore we present the overall mean (between-persons) of the within-person mean MCS score. This was estimated as follows:

1. Calculating the mean MCS score for each participant i (ȳ_i_ according to disability status x_d_ where d = 0 for no disability and d = 1 for disability within category j of the covariate z).
  Given x = d, z = j, for k waves,
  $${\bar{y}}_{i}=\overset{12}{\underset{k=1}{\sum }}y_{i}\times \left(\frac{1}{k}\right)$$
  where y_i_ is the MCS score for participant i, and k is the number of observations (or waves) for individual i within strata of x = d and z = j (equation 1).
2. Calculating the mean of within-person means for x = d, z = j
  Given x = d, z = j,
  $$\bar{y}=\sum {\bar{y}}_{i}$$
  where *ȳ*_*i*_ is the mean MCS score for each participant i within strata of x = d and z = j (equation 2).

We used longitudinal linear regression models with fixed-effects estimators to estimate the association between disability acquisition and a person’s MCS score. Coefficients from the models describe average differences in MCS scores between waves in which individuals reported no disability and waves in which they reported disability. Fixed-effects models remove bias from time-invariant confounding [34]. We controlled for age, employment status and household income because they are potential confounders. In this analysis wealth is time-invariant therefore within-person changes are not estimated. To assess whether the association between disability acquisition and mental health varied by pre-existing wealth, we included a product term between disability acquisition and wealth tertiles, and assessed whether there was statistical evidence of an interaction using the P values of the product terms [35]. All analyses were conducted in Stata/SE 12 (StataCorp, College Station) [36], using the xtreg command with fixed-effects estimators and robust standard errors to fit regression models and the lincom command to compute effect estimates and 95% confidence intervals for each category of wealth. The data used in this paper were extracted from HILDA using the Add-On package PanelWhiz for Stata [37]. Data access and management are administered by the Melbourne School of Population and Global Health, which has signed an Organisational Deed of Licence with The Department of Social Services, enabling academics to use the General Release HILDA dataset for the purpose of research. The HILDA data used in this analysis were anonymised to ensure confidentiality.

### Sensitivity analyses

We conducted the following sensitivity analyses to test the robustness of our findings.

1. Exclusion of people with psychological impairments because the MCS score will be lower and it is possible that the relationships between disability, wealth and mental health may differ;
2. Exclusion of people with imputed wealth data (complete case analysis);
3. Analyses using quintiles (instead of tertiles) of wealth to determine whether this categorisation revealed a different pattern of association; and
4. Analyses including assets and debts in separate models to test the impact of each on the association between disability acquisition and mental health.

## Results

**Fig 1:**
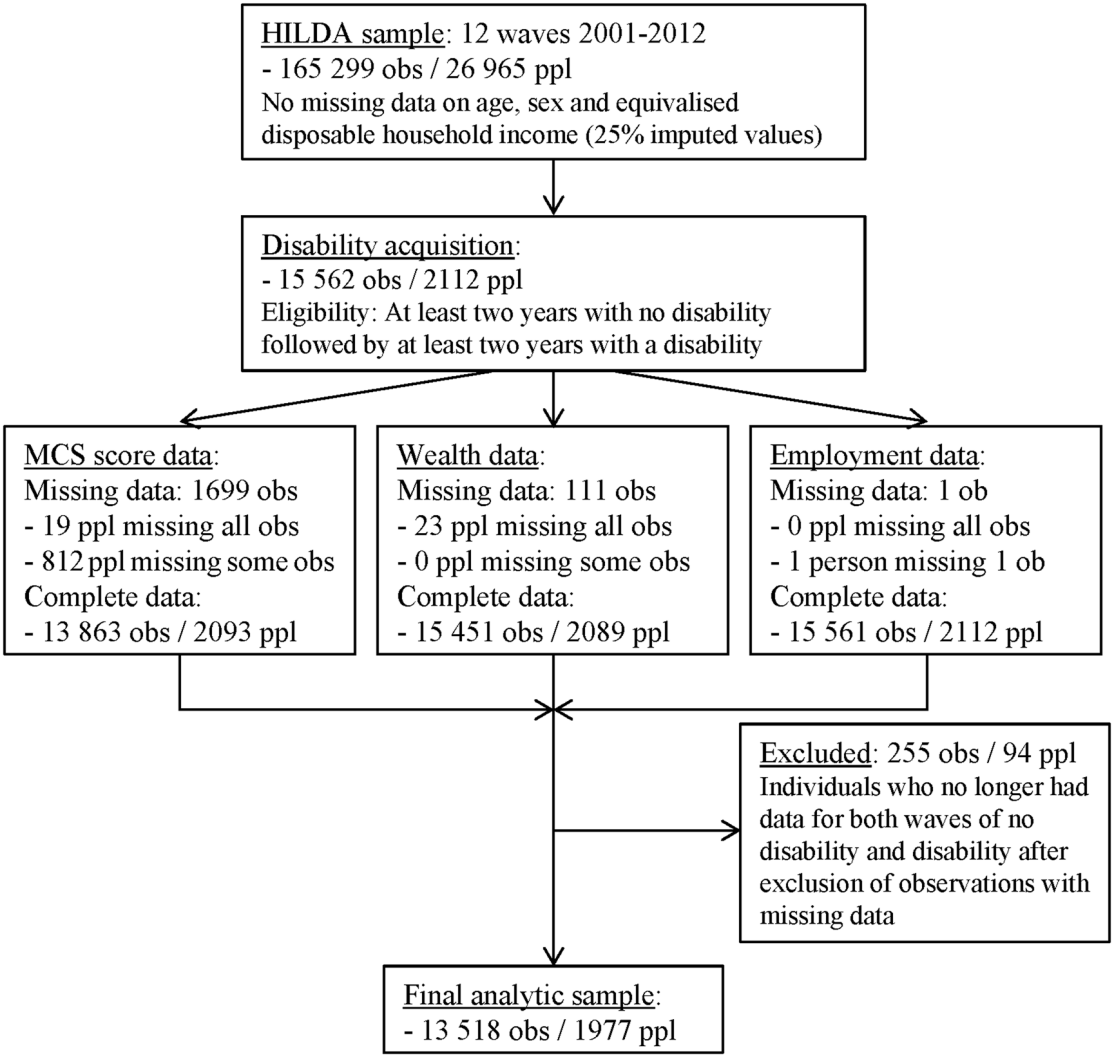
Flow diagram of sample selection and missing data (MCS, mental component summary; obs = observations; ppl = people).

There were 2112 persons (15 562 observations) who met our criteria for disability acquisition. There were complete data available for 98% of people, resulting in a final analytic sample of 1977 persons (14 039 observations). The mean number of observations (contributed annual waves of data) per person was 6.8. Further details of sample selection and missing data are presented in the flow diagram (Fig 1).

At baseline entry into the analytic sample, nearly a third of the sample were aged 60 years or older; 56% were employed and only one in eight were in the highest income quintile while nearly a third were in the lowest quintile. In terms of wealth, 37% were in the highest wealth tertile and 29% the lowest; 41% were in lowest debt tertile; and assets were evenly spread across the tertiles (Table 1).

**Table 1:** Characteristics of sample at baseline entry into the analytic sample (n = 1977 persons).

		n	%
**Age group (years)**	<30	313	15
	30–44	495	25
	45–60	547	28
	60+	622	31
**Sex**	Women	1119	57
	Men	858	43
**Wealth**	High	726	37
	Medium	681	34
	Low	570	29
**Assets**	High	684	35
	Medium	683	35
	Low	610	31
**Debt**	High	538	27
	Medium	630	32
	Low	809	41
**Employment**	Unemployed	63	3
	NILF	785	40
	Low skilled	301	15
	Medium skilled	405	20
	High skilled	423	21
**Income**	High	253	13
	Q4	321	16
	Q3	377	19
	Q2	405	20
	Low	621	31

Table 2 shows the mean of the pooled within-individual MCS scores by disability status for each covariate (see Methods). The MCS score was lower when disability was reported than at baseline (no disability). There were obvious socio-economic gradients with lower mental health in the most disadvantaged groups (unemployed, high debt, low assets and low wealth). The MCS score was positively associated with age and men reported higher mental health scores than women.

**Table 2:** Mean of participants’ pooled MCS score in waves reporting disability and no disability and mean differences between the pooled scores, with 95% confidence intervals (n = 1977, 13 518 observations).

		No disability	Disability
**Whole sample**		49.0 (48.6, 49.4)	47.1 (46.6, 47.5)
**Age group (years)**	<30	44.5 (43.5, 45.6)	40.7 (39.1, 42.4)
	30–44	46.5 (45.7, 47.3)	42.9 (41.9, 44.0)
	45–60	48.7 (48.0, 49.3)	46.5 (45.7, 47.3)
	60+	52.9 (52.4, 53.4)	50.6 (50.0, 51.2)
**Sex**	Women	48.3 (47.7, 48.8)	46.2 (45.6, 46.9)
	Men	49.9 (49.4, 50.5)	48.2 (47.5, 48.8)
**Wealth**	High	50.3 (49.7, 50.9)	49.2 (48.5, 49.9)
	Medium	49.2 (48.6, 49.9)	47.6 (46.8, 48.3)
	Low	47.0 (46.2, 47.8)	43.8 (42.9, 44.8)
**Assets**	High	50.3 (49.7, 50.9)	49.1 (48.4, 49.8)
	Medium	48.9 (48.2, 49.5)	47.2 (46.5, 48.0)
	Low	47.7 (46.9, 48.4)	44.6 (43.7, 45.5)
**Debt**	High	47.9 (47.1, 48.6)	46.4 (45.5, 47.2)
	Medium	47.4 (46.7, 48.1)	45.0 (44.1, 45.8)
	Low	51.0 (50.4, 51.6)	49.2 (48.5, 49.9)
**Employment**	Unemployed	43.2 (41.2, 45.1)	39.7 (37.3, 42.2)
	NILF	50.1 (49.5, 50.7)	47.2 (46.6, 47.9)
	Low skilled	48.2 (47.3, 49.0)	45.1 (43.9, 46.3)
	Medium skilled	47.9 (47.2, 48.6)	46.2 (45.3, 47.1)
	High skilled	48.7 (48.0, 49.4)	47.8 (46.9, 48.7)
**Income**	High	49.5 (48.6, 50.3)	47.6 (46.5, 48.6)
	Q4	48.5 (47.7, 49.3)	47.4 (46.5, 48.3)
	Q3	49.0 (48.3, 49.7)	46.8 (46.0, 47.7)
	Q2	48.3 (47.6, 49.1)	46.8 (45.9, 47.6)
	Low	49.1 (48.4, 49.7)	46.5 (45.7, 47.3)

### Regression analyses

There was statistical evidence to support the inclusion of a product term between disability acquisition and wealth. In the adjusted models the coefficients for the mid and lowest tertiles of wealth were -0.6 (95% CI -1.5, 0.3, p = 0.164) and -2.2 (95% CI -3.1, -1.2, p = <0.001) respectively. Therefore all results were reported across strata of wealth. While there was deterioration in mental health following acquisition of disability for all wealth tertiles, the difference was the greatest in the lowest tertile. There is more than a 2 point difference in the change in MCS score between the highest (-1.1, 95% CI -1.7, -0.5) and lowest tertiles (-3.3, 95%CI -4.0, -2.5) (Table 3).

**Table 3:** Linear fixed-effects regression coefficients for the difference in MCS score within-persons between waves reporting disability and no disability (n = 1977, observations = 13 518) for wealth tertiles separately, adjusted for age, employment and equivalised household disposable income.

	Coeff.	95% CI	P value
**High wealth**	-1.1	-1.7, -0.5	0.001
**Medium wealth** ^a^	-1.7	-2.4, -1.1	<0.001
**Low wealth** ^b^	-3.3	-4.0, -2.5	<0.001

^a^ Interaction term/relative excess risk due to interaction: medium wealth (-0.6, 95% CI -1.5, 0.3, p = 0.164)

^b^ Interaction term/relative excess risk due to interaction: low wealth (-2.2, 95% CI -3.1, -1.2, p<0.001)

Results of our main analyses were robust to sensitivity analyses. Exclusion of people with psychological impairments attenuated the results slightly although the wealth gradient was still evident; the reduction in MCS in the lowest wealth tertile was 2.5 (95% CI -3.2, -1.7) compared to 0.6 (95% CI -1.2, -0.1) among people in the highest wealth tertile (S1 File). The results of the complete case analysis yielded almost identical results (S2 File). When wealth was categorised into quintiles, the strongest effect estimates for disability acquisition on MCS score were in the lowest two wealth quintiles Table C in S3 File). Finally, separate analyses of assets and debt demonstrated that, while people in all tertiles of assets experienced a decrease in MCS score after acquisition of disability, the effect estimates were largest in the lowest tertile (-3.0, 95% CI -3.8, -2.3) (S4 File). The magnitude of the difference in MCS score after disability acquisition was similar for the mid and lowest tertiles of debt (mid -2.4, 95% CI -3.1, -1.7; lowest -1.8, 95% CI -2.4, -1.3) (S5 File).

## Discussion

We found that acquisition of a disability in adulthood was associated with deterioration in mental health. Those in the lowest wealth tertile experienced the greatest deterioration, with a greater than three point decline in MCS score compared to a 1.1 point decline for those in the highest wealth tertile. There was evidence of a negative additive interaction between wealth and disability with relative excess risk due to being in the lowest wealth tertile of about 2 points.

In this paper we used fixed-effects regression to examine within-person change in mental health—an approach we and others have used in a number of other studies of psychosocial working conditions [27], employment arrangements [38], housing affordability [39], and job quality [40,41] in HILDA. The effect estimates for other exposures have been in the range of 1–2.5 points on the MCS scale. In this paper, people with low wealth prior to disability acquisition had a 3.3 point reduction in their mental health, highlighting the importance of these findings. This reduction is particularly important given that this group also had the poorest mental health at baseline (see Table 2).

Our findings are consistent with those of Smith et al. (2005) who found that having net wealth below the median was associated with a greater reduction in subjective wellbeing following acquisition of disability. However, their study was restricted to five waves of data collected at two year intervals in which only 478 people acquired a disability. Further, disability acquisition was defined as one wave without a disability followed by one wave with disability, and thus it is likely the sample included people with transient disabilities [14].

The relationship between wealth and mental health may be driven by assets, debts or both. In HILDA assets and debts are positively correlated (r = 0.45) as wealthy households can more readily obtain loans [16]. In separate analyses of assets and debt we found that those who had the least assets had the greatest deterioration in mental health, with a similar effect estimate as that found for wealth. For debt, a more modest reduction was found in both the mid and lowest tertiles of debt. What remains to be understood are the relative effects of different forms of assets or debt. The main forms of assets in Australia are housing, equities and superannuation, while housing and credit cards are the biggest sources of debt [16]. We have previously shown in the same dataset that low income private renters have poorer mental health than low income home purchasers [42]. It is possible that having equity in a home is an important buffer when a disability is acquired.

It is possible that the impact of wealth varies according to the type of impairment people acquired. It was not possible to investigate this in this study. Although HILDA does collect information on the limitations people experience from the third wave onwards (e.g. sight problems not corrected by glasses, difficulty gripping things, limited use of legs or feet, difficulty learning and understanding things, mental illness or nervous condition). Using these questions respondents can be classified as having sensory and speech, physical, psychological, intellectual and other impairments. A large proportion of people were classified as having more than one impairment type making it difficult to separate out the effects of the different impairment types. Overall the most common type of impairment was physical (70%) followed by sensory and speech (30%); psychological (17%); intellectual (4%) and other (70%).

This study has a number of strengths. First, it is based on a large population-based longitudinal survey in which over 2000 people acquired disability over 12 years. Second, causally-robust fixed-effects regression models were used to control for time-invariant confounding and important time-varying confounders were adjusted for [34]. Third, we used a comprehensive measure of household wealth and were able to examine assets and debt separately.

There are also potential limitations including the amount of missing wealth data. Results from analyses of data with imputed wealth and complete case analysis were nearly identical. Another potential problem is that wealth may have been measured up to three years before the disability status and mental health. While wealth may be more stable than income, the recent volatility in the value of the major household assets—housing, equities and superannuation [16]–may mean that the accuracy of the wealth variable reduces as the time between the measurement of wealth and disability increases. It is likely that any measurement error due to this would be non-differential. Further limitations include the possibility of dependent misclassification bias which is more likely in studies where there are two or more subjectively reported variables (e.g. disability and mental health). The fixed-effects analysis mitigates against this as any time-invariant influences (e.g., negative affectivity) is controlled for. Attrition bias is another potential limitation, but loss to follow-up in HILDA was low (<10% for most waves) [26]. Additionally, HILDA may under-represent people with more severe disability for whom wealth may be more even more important. Finally, the results may not be transportable to other countries. Australia has a Disability Support Pension for people with disabilities who are unable to work which may buffer against some of the negative mental health effects of acquiring a disability. Research in other countries is required in order to investigate the generalizability of these results. In this analysis we investigated effects of disability acquisition on mental health averaged over all consecutive waves of data in which individuals reported a disability (on average three waves of data reporting disability, but up to 10 waves). It is possible that these effects vary over time and that an average effect would not fully explain the patterns of mental health subsequent to disability onset. However, a longitudinal analysis of disability acquisition and subjective wellbeing using the German Socio-Economic Panel and the British Household Panel Survey found no evidence of adaptation over time, indicating that participants maintained lower levels of wellbeing long after the onset of disability [8]. Future research could further examine whether these mental health effects change over time, using methods such as growth curve models or fixed-effects models with lagged effects.

The results of this study suggest that policies and programs should be developed so as to alleviate the additional mental health burden of acquiring a disability among individuals with low wealth.

## Supporting Information

S1 File Supplementary Table A. Linear fixed-effects regression coefficients for the difference in MCS score within-persons between waves reporting disability and no disability for wealth tertiles separately, adjusted for age, employment and equivalised household disposable income—people with psychological impairments excluded (n = 1912, observations = 13,105).
(DOCX)

S2 File Supplementary Table B. Linear fixed-effects regression coefficients for the difference in MCS score within-persons between waves reporting disability and no disability for wealth tertiles separately, adjusted for age, employment and equivalised household disposable income—complete case analysis (sample restricted to non-imputed wealth data) (n = 1594, observations = 11,034).
(DOCX)

S3 File Supplementary Table C. Linear fixed-effects regression coefficients for the difference in MCS score within-persons between waves reporting disability and no disability, adjusted for age, employment and equivalised household disposable income—for quintiles of wealth (n = 1977, observations = 13,518).
(DOCX)

S4 File Supplementary Table D. Linear fixed-effects regression coefficients for the difference in MCS score within-persons between waves reporting disability and no disability for tertiles of assets separately, adjusted for age, employment and equivalised household disposable income—assets (n = 1977, observations = 13,518).
(DOCX)

S5 File Supplementary Table E. Linear fixed-effects regression coefficients for the difference in MCS score within-persons between waves reporting disability and no disability for tertiles of debt separately, adjusted for age, employment and equivalised household disposable income—debt (n = 1977, observations = 13,518).
(DOCX)
