# Supplementary material for: Mental Health Following Acquisition of Disability in Adulthood—The Impact of Wealth
Source: PLoS One. 2015 Oct 7;10(10):e0139708. doi: 10.1371/journal.pone.0139708 (PMC4596479; doi:10.1371/journal.pone.0139708)
Supplement: S1 File — Linear fixed-effects regression coefficients for the difference in MCS score within-persons between waves reporting disability and no disability for wealth tertiles separately, adjusted for age, employment and equivalised household disposable income—people with psychological impairments excluded (n = 1912, observations = 13,105). (DOCX) [file pone.0139708.s001.docx]

Supplementary Table A. Linear fixed-effects regression coefficients for the difference in MCS score within-persons between waves reporting disability and no disability for wealth tertiles separately, adjusted for age, employment and equivalised household disposable income– people with psychological impairments excluded (n=1912, observations=13,105)

|  | Coeff. | 95% CI | P value |
| --- | --- | --- | --- |
| **High wealth** | -0.6 | -1.2, -0.1 | 0.028 |
| **Medium wealth**^a^ | -1.3 | -1.9, -0.7 | <0.001 |
| **Low wealth**^b^ | -2.5 | -3.2, -1.7 | <0.001 |

^a^ Interaction term/relative excess risk due to interaction: medium wealth (-0.6, 95% CI -1.5, 0.2, p=0.152)

^b^ Interaction term/relative excess risk due to interaction: low wealth (-1.8, 95% CI -2.8, -0.8, p<0.001)
