# Supplementary material for: Mental Health Following Acquisition of Disability in Adulthood—The Impact of Wealth
Source: PLoS One. 2015 Oct 7;10(10):e0139708. doi: 10.1371/journal.pone.0139708 (PMC4596479; doi:10.1371/journal.pone.0139708)
Supplement: S2 File — Linear fixed-effects regression coefficients for the difference in MCS score within-persons between waves reporting disability and no disability for wealth tertiles separately, adjusted for age, employment and equivalised household disposable income—complete case analysis (sample restricted to non-imputed wealth data) (n = 1594, observations = 11,034). (DOCX) [file pone.0139708.s002.docx]

Supplementary Table B. Linear fixed-effects regression coefficients for the difference in MCS score within-persons between waves reporting disability and no disability for wealth tertiles separately, adjusted for age, employment and equivalised household disposable income – complete case analysis (sample restricted to non-imputed wealth data) (n=1594, observations=11,034)

|  | Coeff. | 95% CI | P value |
| --- | --- | --- | --- |
| **High wealth** | -1.1 | -1.8, -0.5 | 0.001 |
| **Medium wealth**^a^ | -1.6 | -2.4, -0.9 | <0.001 |
| **Low wealth**^b^ | -3.3 | -4.2, -2.5 | <0.001 |

^a^ Interaction term/relative excess risk due to interaction: medium wealth (-0.5, 95% CI -1.5, 0.5, p=0.318)

^b^ Interaction term/relative excess risk due to interaction: low wealth (-2.2, 95% CI -3.3, -1.1, p<0.001)
