# Supplementary material for: Mental Health Following Acquisition of Disability in Adulthood—The Impact of Wealth
Source: PLoS One. 2015 Oct 7;10(10):e0139708. doi: 10.1371/journal.pone.0139708 (PMC4596479; doi:10.1371/journal.pone.0139708)
Supplement: S3 File — Linear fixed-effects regression coefficients for the difference in MCS score within-persons between waves reporting disability and no disability, adjusted for age, employment and equivalised household disposable income—for quintiles of wealth (n = 1977, observations = 13,518). (DOCX) [file pone.0139708.s003.docx]

Supplementary Table C. Linear fixed-effects regression coefficients for the difference in MCS score within-persons between waves reporting disability and no disability, adjusted for age, employment and equivalised household disposable income – for quintiles of wealth (n=1977, observations=13,518)

|  | Coeff. | 95% CI | P value |
| --- | --- | --- | --- |
| **High wealth** | -0.8 | -1.5, -0.1 | 0.035 |
| **Q2**^a^ | -2.0 | -2.9, -1.2 | <0.001 |
| **Q3**^b^ | -1.2 | -2.0, -0.4 | 0.003 |
| **Q4**^c^ | -2.7 | -3.6, -1.8 | <0.001 |
| **Low wealth**^d^ | -3.6 | -4.6, -2.5 | <0.001 |

^a^ Interaction term/relative excess risk due to interaction: Q2 wealth (-1.3, 95% CI -2.4, -0.1, p=0.029)

^b^ Interaction term/relative excess risk due to interaction: Q3 wealth (-0.4, 95% CI -1.5, 0.7, p=0.446)

^c^ Interaction term/relative excess risk due to interaction: Q4 wealth (-1.9, 95% CI -3.0, -0.8, p=0.001)

^d^ Interaction term/relative excess risk due to interaction: Q5 wealth (-2.8, 95% CI -4.1, -1.5, p<0.001)
