# Supplementary material for: Mental Health Following Acquisition of Disability in Adulthood—The Impact of Wealth
Source: PLoS One. 2015 Oct 7;10(10):e0139708. doi: 10.1371/journal.pone.0139708 (PMC4596479; doi:10.1371/journal.pone.0139708)
Supplement: S4 File — Linear fixed-effects regression coefficients for the difference in MCS score within-persons between waves reporting disability and no disability for tertiles of assets separately, adjusted for age, employment and equivalised household disposable income—assets (n = 1977, observations = 13,518). (DOCX) [file pone.0139708.s004.docx]

|  | Coeff. | 95% CI | P value |
| --- | --- | --- | --- |
| **High assets** | -1.1 | -1.7, -0.5 | <0.001 |
| **Medium assets**^a^ | -1.7 | -2.4, -1.1 | <0.001 |
| **Low assets**^b^ | -3.0 | -3.8, -2.3 | <0.001 |

Supplementary Table D. Linear fixed-effects regression coefficients for the difference in MCS score within-persons between waves reporting disability and no disability for tertiles of assets separately, adjusted for age, employment and equivalised household disposable income – assets (n=1977, observations=13,518)

^a^ Interaction term/relative excess risk due to interaction: medium assets (-0.6, 95% CI -1.5, 0.3, p=0.172)

^b^ Interaction term/relative excess risk due to interaction: low assets (-1.9, 95% CI -2.9, -1.0, p<0.001)
