# Supplementary material for: Mental Health Following Acquisition of Disability in Adulthood—The Impact of Wealth
Source: PLoS One. 2015 Oct 7;10(10):e0139708. doi: 10.1371/journal.pone.0139708 (PMC4596479; doi:10.1371/journal.pone.0139708)
Supplement: S5 File — Linear fixed-effects regression coefficients for the difference in MCS score within-persons between waves reporting disability and no disability for tertiles of debt separately, adjusted for age, employment and equivalised household disposable income—debt (n = 1977, observations = 13,518). (DOCX) [file pone.0139708.s005.docx]

Supplementary Table E. Linear fixed-effects regression coefficients for the difference in MCS score within-persons between waves reporting disability and no disability for tertiles of debt separately, adjusted for age, employment and equivalised household disposable income – debt (n=1977, observations=13,518)

|  | Coeff. | 95% CI | P value |
| --- | --- | --- | --- |
| **High debt** | -1.4 | -2.2, -0.7 | <0.001 |
| **Medium debt**^a^ | -2.4 | -3.1, -1.7 | <0.001 |
| **Low debt**^b^ | -1.8 | -2.4, -1.3 | <0.001 |

^a^ Interaction term/relative excess risk due to interaction: medium debt (-1.0, 95% CI -2.0, 1.0, p=0.076)

^b^ Interaction term/relative excess risk due to interaction: low debt (-0.4, 95% CI -1.3, 0.6, p=0.439)
